# Bone marrow-derived extracellular vesicles from multiple myeloma patients promote adaptive immune dysfunction via HLA-G, PD-1, and PD-L1

**DOI:** 10.3389/fimmu.2025.1640168

**Published:** 2025-08-28

**Authors:** Debora Soncini, Danilo Marimpietri, Francesco Ladisa, Francesco Lai, Irma Airoldi, Roberto Gramignoli, Michele Cea, Fabio Morandi

**Affiliations:** ^1^ Clinic of Hematology, Department of Internal Medicine (DiMI), University of Genoa, Genova, Italy; ^2^ UOSD Laboratory of Cell Therapies, IRCCS Istituto Giannina Gaslini, Genova, Italy; ^3^ IRCCS Ospedale Policlinico San Martino, Genoa, Italy

**Keywords:** multiple myeloma, extracellular vesicles, immune checkpoints, HLA-G, PD-L1

## Abstract

**Introduction:**

Extracellular vesicles (EVs) are critical mediators of intercellular communication and contribute to cancer progression and immune regulation.

**Methods:**

We characterized EVs isolated from bone marrow (BM) plasma harvested from healthy donors and patients affected by Multiple Myeloma (MM) by Nano Tracking Analysis and by flow cytometry.

**Results:**

EVs from MM patients were significantly more abundant and enriched in CD138, supporting their partial origin from malignant plasma cells, with additional input from BM resident cells, including monocytes and NK cells. Phenotypic profiling revealed increased expression of immune checkpoint molecules HLA-G, PD-1, and PD-L1 on MM-derived EVs compared to healthy controls. Functionally, MM-EVs suppressed Staphylococcal enterotoxin B (SEB)-induced T cell activation, as evidenced by reduced IFN-γ production and CD4^+^ T cell proliferation. Such effects were partially reversed by HLA-G blockade. Moreover, MM-derived EVs modulated cytokine secretion profiles suppressing IL-2, IFN-α, TNF-α, and IL-6, and enhancing GM-CSF, with some changes attributed to HLA-G and PD-L1 activity. Transcriptomic analysis showed higher HLA-G expression in patients with gain of chromosome 1q, suggesting a link between high-risk cytogenetics and EV-driven immune suppression. While clinical correlations were not observed, likely due to limited sample size, these findings underscore the immunosuppressive role of MM-derived EVs.

**Discussion:**

HLA-G^+^, PD-1^+^, and PD-L1^+^ EVs contribute to immune dysfunction in MM and represent promising targets to restore anti-tumor immunity.

## 1 Introduction

Multiple myeloma (MM) is a hematological malignancy primarily affecting individuals over the age of 60 years and accounts for approximately 0.9% of all cancers worldwide and contributes to 1.2% of cancer-related deaths (1). MM is characterized by the clonal expansion of malignant plasma cells (PCs) within the bone marrow (BM). While etiology remains incompletely understood, several cytogenetic abnormalities, including chromosomal deletions and translocations, have been identified as key drivers of disease progression and are frequently associated with high-risk MM (2). MM often arises from asymptomatic precursor conditions such as monoclonal gammopathy of undetermined significance (MGUS) and smoldering multiple myeloma (SMM). Progression to symptomatic MM is typically marked by organ damage, generally manifested as hypercalcemia, renal insufficiency, anemia, and bone lesions (2). Malignant PCs within the BM express various immune checkpoint molecules supporting immune evasion. Among these, HLA-G and PD-L1 have been implicated in suppressing anti-tumor immune responses (3, 4). HLA-G is a non-polymorphic HLA class I molecule, identified in seven isoforms, functioning either as a surface molecule or in soluble form. HLA-G exerts immunomodulatory effects by interacting with inhibitory receptors on T lymphocytes and natural killer (NK) cells (5). Soluble HLA-G has been described as released by patients’ primary malignant PCs and MM cell lines (6, 7), while surface protein and mRNA expression have been confirmed in patient-derived PCs (4, 8). Notably, HLA-G can be transferred from malignant PCs to T cells via trogocytosis, thereby impairing anti-tumor immunity (4). Programmed cell death protein 1 (PD-1) is another inhibitory receptor primarily expressed on activated T cells and, to a lesser extent, on NK cells (9, 10). PD-1 is upregulated during the late stages of T cell activation and functions to suppress immune responses by modulating intracellular signaling upon ligand engagement (3, 4, 11). PD-1 ligands, PD-L1, and PD-L2 have distinct expression patterns: PD-L2 is restricted to antigen-presenting cells, whereas PD-L1 is broadly expressed on both normal and malignant tissues (12, 13). In MM, PD-L1 is upregulated not only on malignant PCs but also in other components of the tumor microenvironment (9, 14, 15). Preclinical and early-phase clinical studies have shown that inhibition of the PD-1/PD-L1 axis can enhance the efficacy of standard MM therapies (13, 16, 17). Tumor-derived extracellular vesicles (EVs) are present in the BM of MM patients and have been shown to participate in various pathogenic processes, including immune suppression, tumor progression, drug resistance, angiogenesis, osteolysis, and metastatic niche formation (18–21). We previously demonstrated that MM-derived EVs are enriched in adenosinergic ectoenzymes (CD38, CD39, CD203a, and CD73), which catalyze the production of extracellular adenosine (ADO), a potent immunosuppressive metabolite (22). These enzymes contribute to shaping the immunosuppressive BM microenvironment by increasing local ADO levels (23, 24). Given the multifaceted role of EVs in MM biology, this study aimed to: i) characterize EVs isolated from BM plasma of MM patients; ii) assess the expression of the immune checkpoint molecules HLA-G, PD-1, and PD-L1 on these EVs; iii) investigate their immunoregulatory functions, with a particular focus on the roles of HLA-G and the PD-1/PD-L1 axis.

## 2 Materials and methods

### 2.1 Patients

BM samples were collected from 42 patients with MM, including newly diagnosed (NDMM, n = 31) and relapsed/refractory (RRMM, n = 11) cases admitted to IRCCS Ospedale Policlinico San Martino. Control BM samples were collected from healthy donors (HD, n = 12) undergoing BM donation. Written informed consent was obtained from all participants in accordance with the Declaration of Helsinki. Clinical characteristics of MM patients are summarized in **Table 1**.

**Table 1:** Patients’ characteristics.

Age at diagnosis	Disease stage	Myeloma subtype	R-ISS-stage	CA
67	NDMM	IgG kappa	III	hyperdiploidy
58	NDMM	IgA lambda,	III	t (4;14), del17 p, amp 1q
85	RRMM	Non-secretory	II	none
64	RRMM	Non-secretory	II	none
77	RRMM	IgG kappa	I	standard
78	NDMM	micromolecular K	I	N/A
49	RRMM	IgG kappa	II	del17p
70	NDMM	IgA lambda	I	none
68	NDMM	IgG kappa	I	N/A
78	NDMM	IgA kappa	I	N/A
75	RRMM	IgG kappa	N/A	N/A
64	RRMM	IgA kappa	III	del17p
77	NDMM	IgG lambda	II	none
81	NDMM	IgG lambda	I	N/A
82	NDMM	IgA kappa	II	none
55	NDMM	IgG kappa	I	none
84	NDMM	IgG lambda	II	none
64	NDMM	IgG kappa	II	none
65	NDMM	IgA lambda	III	none
78	NDMM	IgG kappa	II	none
65	NDMM	IgG kappa	I	none
62	NDMM	IgA lambda	III	amp1q
85	RRMM	Non-secretory	II	none
70	NDMM	IgG kappa	III	none
70	RRMM	IgG kappa	I	none
79	NDMM	IgG kappa	I	none
78	NDMM	IgG lambda	III	del17p
70	NDMM	micromolecular K	I	t (4;14)
82	NDMM	IgG kappa	I	none
71	RRMM	IgG kappa	II	del17p; t (14;16)
70	RRMM	IgA kappa	III	none
75	NDMM	IgA kappa	III	del17p
78	NDMM	micromolecular K	I	none
50	NDMM	IgA lambda	III	t (4,14)
85	NDMM	IgG lambda	I	N/A
81	NDMM	IgG Kappa	I	N/A
76	NDMM	IgG Kappa	I	none
62	NDMM	IgG Kappa	I	none
65	NDMM	IgG Kappa	III	t (14;16), 1q amp
76	RRMM	IgG Kappa	I	N/A
61	NDMM	micromolecular lambda	III	t (4;14) 1qamp
60	NDMM	IgG kappa	II	t (14;16)

NDMM, newly diagnosed multiple myeloma; RRMM, relapsed/refractory multiple myeloma; CA, chromosomal abnormalities; N/A, not available.

### 2.2 Isolation and characterization of EV

BM plasma was obtained by centrifugation of BM blood at 3,000 × g for 15 minutes at 4°C. One plasma aliquot (300 μL), or two plasma aliquots (for a small number of patients) were stored at –80°C until analysis. EVs were isolated as previously described (24). Briefly, plasma was diluted 1:3 in PBS and centrifuged at 3,000 × g for 15 minutes at 4°C to remove platelets and debris. Supernatants were collected and analyzed using a NanoSight NS500 (NTA 2.3 software, 488 nm laser) to determine EV size and concentration. Subsequently, EVs were pelleted by centrifugation at 20,000 × g for 1 hour at 4°C, washed with PBS, and resuspended in 50 μL MACS buffer (PBS/EDTA with 0.5% BSA). For functional experiments, EVs were resuspended in serum-free RPMI-1640 under sterile conditions.

### 2.3 Flow cytometric analysis

EV obtained from 300 μL of BM plasma samples were used for flow cytometric analysis. The number of EV used for flow cytometry was variable among samples from patients and controls. The cellular origin of BM-derived EVs was assessed on a small number of MM patients (n=10) depending on the availability of two different aliquots of BM plasma. This analysis was performed using monoclonal antibodies (mAbs) directly conjugated with fluorochromes, targeting CD45 (FITC, BD), CD34 (PE, Miltenyi Biotec), HLA-DR (APC, BD), CD3 (PE-CF594, BD), CD19 (FITC, BD), CD105 (PE, BD), and CD14 (PC5, Beckman Coulter). CD38 (APC, BD) was included as a marker of malignant PCs. Immune checkpoint molecules were evaluated on the whole cohort of patients and controls using anti-HLA-G (PE, BioLegend, clone 87G), anti–PD-1 (Alexa Fluor 488, BioLegend, clone NAT105), and anti–PD-L1 (PE/Cy7, BioLegend, clone 29E2A3). CD138 expression was analyzed using rat anti-CD138 (Alexa Fluor 488, R&D Systems, clone 359103). EVs were incubated with mAbs for 20 minutes in the dark at 4°C, washed, centrifuged at 20,000 × g for 1 hour at 4°C, and resuspended in 200 μL MACS buffer. Flow cytometric analysis was performed on a Gallios cytometer (Beckman Coulter) and analyzed using Kaluza software. A minimum of 2,000 events were acquired per sample using appropriate gating based on forward and side scatter parameters to exclude debris and background. Results were expressed as mean relative fluorescence intensity (MRFI) or percentage of positive EVs for the indicated markers.

### 2.4 Functional studies

Buffy coats from healthy donors (n = 3) were obtained from IRCCS San Martino. Mononuclear cells (MNCs) were isolated via Ficoll-Paque density gradient centrifugation (2,000 × g, 30 min), and seeded into 96-well plates (1 × 10^6^ cells/well) in RPMI-1640 supplemented with 5% human AB serum. To achieve a sufficient number of EVs for functional assays, we isolated them from pooled BM plasma of 4 MM patients, including 2 NDMM and 2 RRMM cases. The pooled EVs were subsequently split into three treatment groups: untreated, pre-treated with anti–HLA-G mAb, or pre-treated with anti–PD-L1 mAb. EVs (200 × 10^6^ particles/well) were pre-incubated with blocking antibodies (10 μg/well) for 30 minutes at room temperature before being added to MNCs. Control wells contained MNCs without EVs. Plates were incubated overnight at 37°C, 5% CO_2_.

### 2.5 IFN-γ secretion assay

IFN-γ secretion was measured using the Rapid Cytokine Inspector kit (Miltenyi Biotec) according to the manufacturer’s instructions. After overnight incubation, MNCs were stimulated with SEB (2 μg/mL, Sigma Aldrich) for 2 hours, followed by the addition of Brefeldin A (1:5 dilution). Cells were then incubated for an additional 4 hours. After removal of supernatant, staining solution containing anti–IFN-γ mAb was added. Fixation and permeabilization steps were performed, and cells were washed and analyzed using a MACSQuant 10 cytometer. Results were expressed as the percentage of IFN-γ^+^ cells among CD3^+^CD4^+^ and CD3^+^CD8^+^ T cells.

### 2.6 T cell proliferation assay

T cell proliferation was assessed using the CellTrace™ CFSE Cell Proliferation Kit (Thermo Fisher Scientific). MNCs were stained with CFSE (2 μM, 15 min, 37°C), washed, and resuspended in RPMI-1640 with 5% AB serum. Cells were incubated overnight with or without EVs and stimulated with SEB (1 μg/mL) for 6 days. Non-stimulated wells served as controls. After incubation, supernatants were stored at –20°C. Cells were stained with anti-CD3 (PC7), anti-CD4 (APC), and anti-CD8 (PE) for 20 minutes at room temperature, washed, and analyzed using a Gallios cytometer. Results were expressed as the percentage of proliferating CD3^+^CD4^+^ and CD3^+^CD8^+^ T cells based on CFSE dilution.

### 2.7 Cytokine secretion

Cytokine concentrations in supernatants collected from the proliferation assay (after 6 days of culture) were measured using the MACSPlex Cytokine 12 kit (Miltenyi Biotec), following the manufacturer’s instructions. This bead-based multiplex assay quantified GM-CSF, IFN-α, IFN-γ, IL-2, IL-4, IL-5, IL-6, IL-9, IL-10, IL-12p70, IL-17A, and TNF-α. Supernatants were centrifuged (10,000 × g, 10 min, 4°C), incubated with capture beads for 2 hours, followed by detection reagent incubation for 1 hour. After washing, samples were acquired on the MACSQuant 10 using the specific “Express Mode.” Cytokine levels were expressed in pg/mL.

### 2.8 Transcriptomic analysis

To complement phenotypic data, transcriptomic analyses were performed using publicly available RNA-sequencing data from the Multiple Myeloma Research Foundation (MMRF) CoMMpass study (IA18 release). Gene expression levels in CD138^+^ plasma cells were analyzed with respect to cytogenetic subgroups, including patients with or without translocation in t(4;14) or gain of 1q abnormalities. Expression data were retrieved in TPM (transcripts per million) format and log_2_-transformed for comparative analyses. Statistical correlations between gene expression and cytogenetic features were assessed using appropriate non-parametric tests.

### 2.9 Statistical analysis

Statistical analysis was performed using GraphPad Prism v5.03. Data normality was assessed using the D’Agostino–Pearson omnibus test. Parametric (t-test) or non-parametric (Mann–Whitney) tests were applied accordingly. Correlations were assessed using linear regression and Spearman’s r. p values < 0.05 were considered statistically significant. Correlograms were generated in R v4.4.1 using RStudio v2024.12.0 + 467.

## 3 Results

### 3.1 Extracellular vesicle characterization in bone marrow samples from MM patients and healthy donors

**Figure 1:**
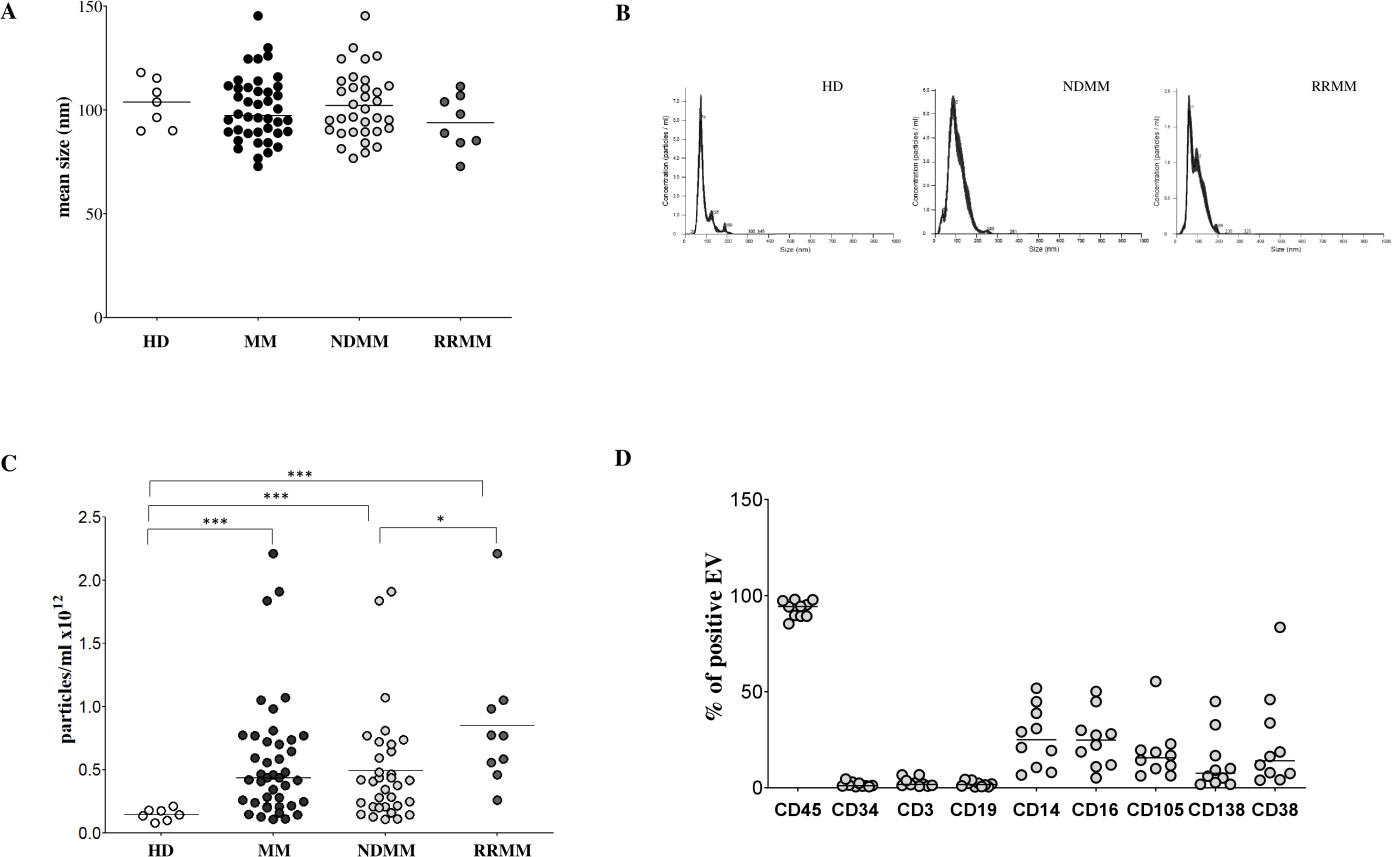
Characterization of EV in BM samples. The size of EVs was analyzed in BM samples harvested from HDs (white dots) and MM patients (black dots), including newly diagnosed (ND; light grey dots) and relapsed/refractory (RR; dark grey dots) subgroups **(A)**. A representative size distribution (in nanometers, nm) for HDs and MM patients (ND, RR) is shown in panel **(B)** The analysis of EV concentration in BM samples from HD (white dots) and MM patients (black dots), including newly diagnosed (ND; light grey dots) and relapsed/refractory (RR; dark grey dots) subgroups is shown in Panel **(C)** Results are expressed as particles/ml x 10^12^. Expression of markers representative of BM-resident cell subsets (CD45, CD34, CD3, CD19, CD14, CD16, CD105 and CD38) on EVs isolated from BM samples of 10 MM patients, expressed as % of positive EVs is shown in Panel **(D)** Horizontal bars indicated medians. Statistically significant differences are indicated (ns p > 0.05; *p ≤ 0.05; **p ≤ 0.01; ***p ≤ 0.001).

We characterized EVs isolated from BM plasma of 42 MM patients, comprising 34 NDMM and 8 RRMM cases, as well as 7 HDs. EVs were analyzed using NTA to assess size distribution and concentration. As shown in **Figure 1A**, the mean EV size was comparable across groups: MM patients (mean ± standard error [SE]: 97.3 ± 2.43 nm) and healthy donors (103.8 ± 4.34 nm), with no significant differences observed between NDMM (98.55 ± 2.76 nm) and RRMM (93.4 ± 4.68 nm) subgroups. A representative EV distribution for healthy donors, NDMM and RRMM is shown in **Figure 1B**. In contrast, EV concentration changed significantly among groups (**Figure 1C**). HDs exhibited a significantly lower EV concentration (mean ± standard deviation [SD]: 0.14 ± 0.01 × 10¹² particles/ml) compared to MM patients overall (0.43 ± 0.07 × 10¹² particles/ml, p = 0.0004). Such increase of EV was confirmed also in NDMM (0.41 ± 0.07 × 10¹² particles/ml, p = 0.0009) and RRMM (0.77 ± 0.18 × 10¹² particles/ml, p = 0.0002) patients, with a significantly higher EV concentration in RRMM group (p = 0.01), suggesting a potential association between disease progression and increased EV release. Together, these findings indicate that EV concentration is markedly elevated in MM patients, particularly in relapsed/refractory cases, while EV size remains consistent across healthy and diseased individuals. This highlights the potential of EV concentration as a biomarker for disease activity, progression, and possibly treatment monitoring in MM.

### 3.2 Immunophenotypic profiling of bone marrow-derived EVs reveals monocyte, NK cell, and plasma cell contributions in MM patients

**Figure 2:**
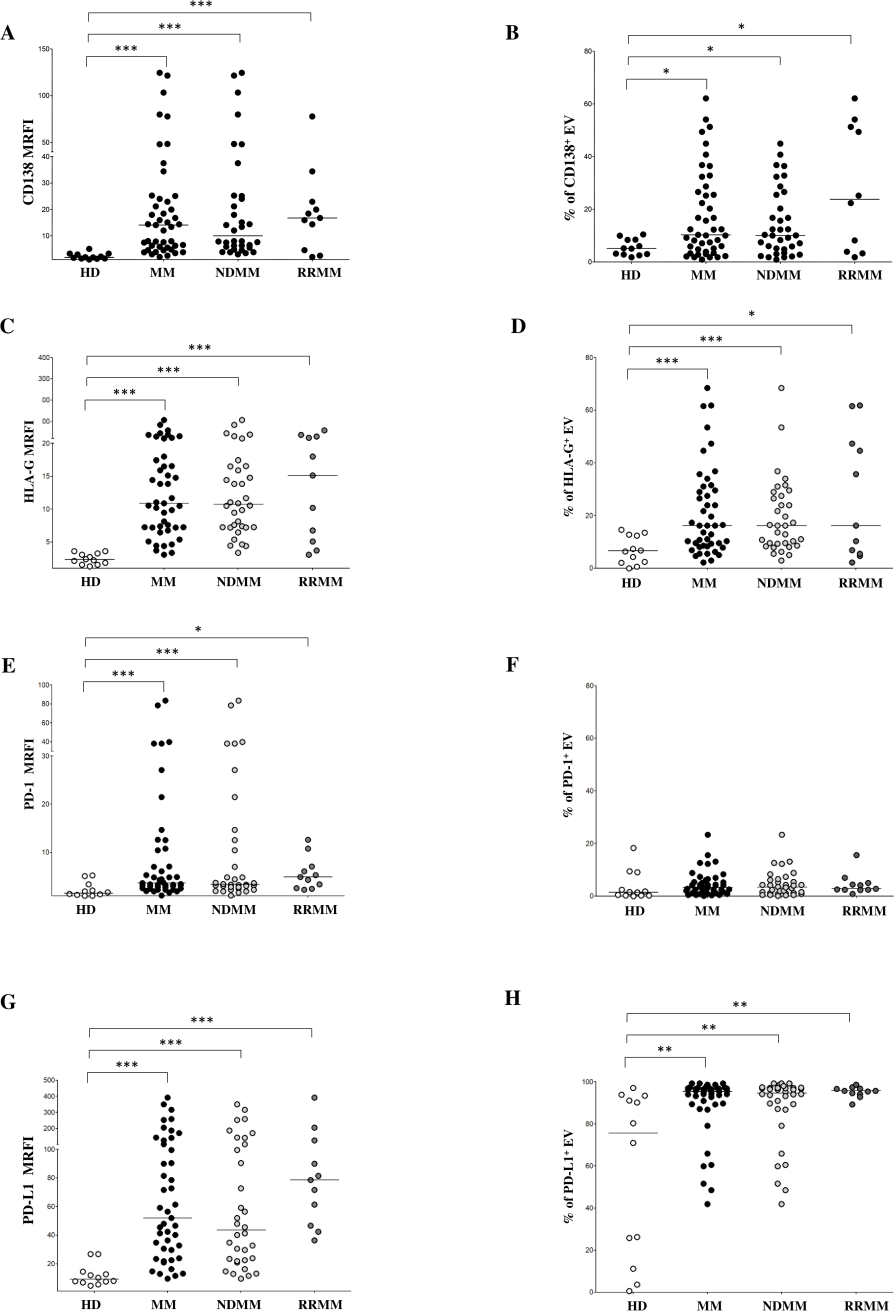
Phenotypical analysis of EVs in BM samples. Expression of CD138 **(A, B)**, HLA-G **(C, D)**, PD-1 **(E, F)** and PD-L1 **(G, H)** on EVs from HD (grey dots) and MM patients (black dots), including ND (grey dots) and RR (black dots) patients. Results are expressed as median relative fluorescence intensity (MRFI) and percentage of CD138^+^ EVs. Horizontal bars indicated medians. Statistically significant differences are indicated. (ns p > 0.05; *p≤ 0.05; **p≤ 0.01; ***p≤ 0.001).

To investigate the BM EV cellular origin, we performed flow cytometric profiling on bone marrow plasma-derived EVs from selected MM patients (n = 10) using a panel of lineage-specific surface markers. As shown in **Figure 1D**, the majority of EVs expressed CD45 (median ± SE: 94.3 ± 1.38%), indicating a predominant leukocyte-derived origin. In contrast, CD34 expression was minimal (1.1 ± 0.4%), effectively excluding the hematopoietic stem cells as origin. To further delineate the contributions from distinct immune cell subsets, we evaluated markers including CD3 (T cells), CD19 (B cells), CD14 (monocytes), and CD16 (NK cells). CD16 can also be expressed by neutrophils and a subset of monocytes and macrophages, but it is usually expressed by NK cells at the highest levels. CD3 and CD19 expression was low (1.7 ± 0.71% and 1.7 ± 0.43%, respectively), whereas CD14 and CD16 showed notably high expression (25 ± 4.9% and 24.8 ± 4.5%, respectively), suggesting that monocytes and NK cells are significant contributors to EV production in MM, while T and B cell contribution on such regard is comparatively minor. Interestingly, we detected moderate expression of CD105 (15.59 ± 4.48%), typically associated with stromal cells. However, CD105 co-expression with CD45 suggests a leukocyte origin, likely from endothelium-associated monocytes or M2-like macrophages, rather than stromal cells. Such results support a prominent role in EV generation played by monocyte/macrophage lineage. PCs were also identified as an active source of EVs, as indicated by CD138 expression (7.6 ± 4.6%). We further analyzed CD38 expression on EVs, an established marker of myeloma cells and other immune subsets such as NK cells and monocytes. CD38 expression was heterogeneous (14 ± 7.9%), consistent with a diverse cellular origin for EVs within the BM microenvironment. To specifically evaluate EVs derived from malignant PCs, we measured CD138 expression across the entire MM cohort and HDs. As shown in **Figure 2A**, CD138 expression was significantly lower in HD-EVs (CD138 MRFI, median ± SE: 1.85 ± 0.33) compared to MM patients (14.08 ± 4.56, p < 0.0001), including both NDMM (10.01 ± 5.71, p < 0.0001) and RRMM (16.76 ± 6.18, p < 0.0001) subgroups. No significant difference was observed between NDMM and RRMM patients. Similarly, the percentage of CD138^+^ EVs was significantly lower in HDs (5.13 ± 0.88%) compared to whole MM patients (10.30 ± 2.47%, p = 0.02) and subgroups NDMM (10.19 ± 2.2%, p = 0.01), and RRMM (22.35 ± 7.1%, p = 0.02), again without a significant difference between disease stages (**Figure 2B**). Collectively, these results validate that MM-derived EVs are predominantly released by leukocytes, particularly monocytes and NK cells, in the bone marrow milieu, with a substantial contribution from malignant plasma cells. These data underscore the potential of EVs as disease-specific biomarkers and key mediators of tumor–microenvironment interactions in MM.

### 3.3 Expression of immune checkpoint molecules is increased in EVs from MM patients

**Figure 3:**
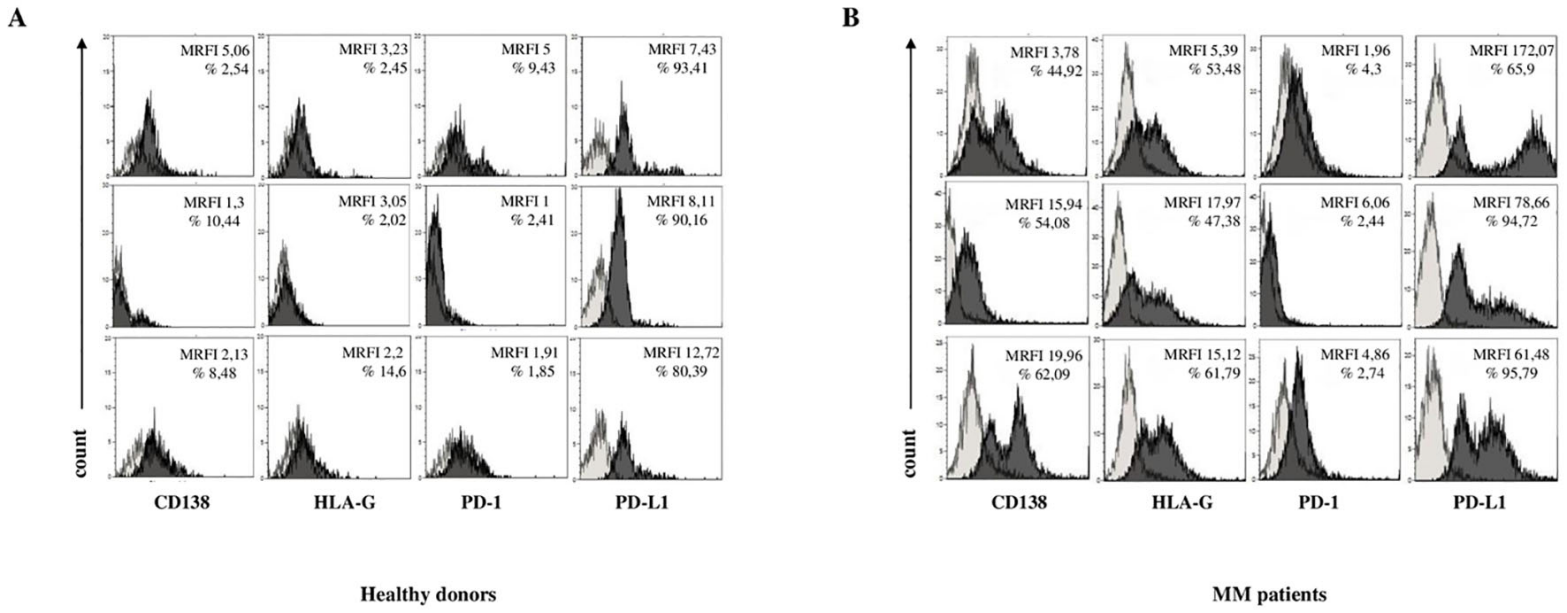
Phenotypical analysis of EV in BM samples. Representative flow cytometry profiles for each marker in three HDs **(A)** and three MM patients **(B)**. Light grey profiles indicate isotype-matched controls, and dark grey profiles represent specific antibody staining.

**Figure 4:**
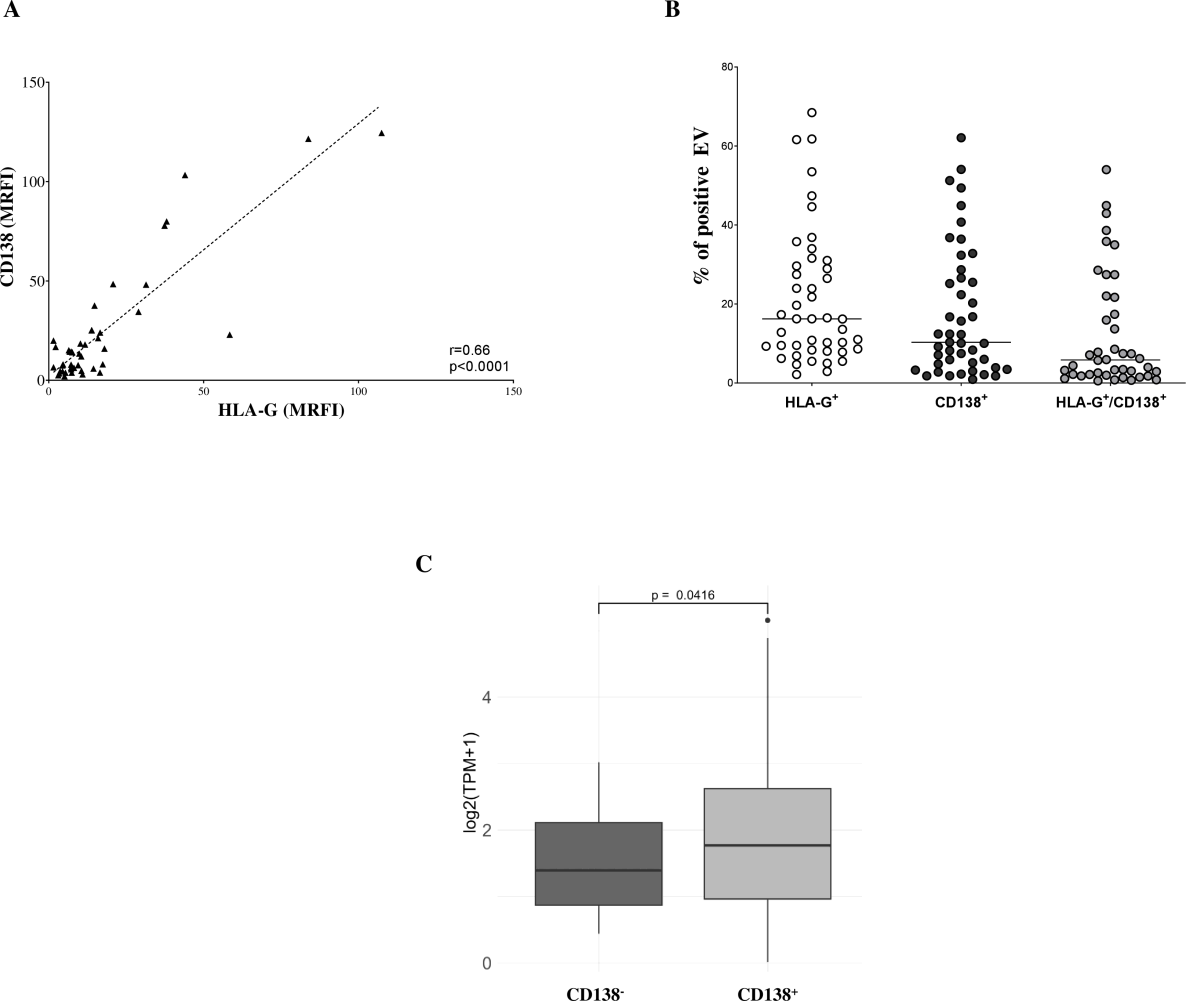
Phenotypic analysis of EV in BM samples. **(A)** Correlations and linear regressions between CD138 and HLA-G expression. **(B)** Percentages of EVs expressing CD138, HLA-G, or co-expressing both molecules. Horizontal bars indicated medians. **(C)** HLA-G transcript levels in MM plasma cells and BM healthy counterparts expressed as log2(TPM+1). Box plots indicated first and third quartiles and medians, whereas whiskers indicated maximum and minimum values. P values (t-test) and correlation values (Pearson’s) are indicated.

To assess the immunoregulatory potential of MM-derived EVs, we analyzed the surface expression of HLA-G, PD-1, and PD-L1 via flow cytometry. As shown in **Figure 2C**, HLA-G expression levels (measured by MRFI) were significantly higher in EVs isolated from MM patients (MRFI, median ± SE: 10.91 ± 3.08) compared to HDs (2.29 ± 0.23, p < 0.0001). Similar values were measured in NDMM (10.72 ± 3.76, p < 0.0001) and RRMM (15.12 ± 5.16, p < 0.0001) subgroups, both significantly elevated over controls (p<0.0001). The percentage of HLA-G^+^ EVs followed a similar trend (**Figure 2D**) with MM patients showing increased frequencies (16.24 ± 2.53%) relative to HDs (6.59 ± 1.52%, p = 0.001). Again, both NDMM (16.23 ± 2.44%, p = 0.0006) and RRMM (16.24 ± 7.13%, p = 0.01) were significantly higher than controls, without significant differences between the disease stages. PD-1 expression, as assessed by MRFI, was also significantly elevated in MM-derived EVs (3.65 ± 2.7) compared to HDs (1.53 ± 0.44, p = 0.0005), with no notable differences between NDMM (3.35 ± 3.52) and RRMM (4.86 ± 1.02) (**Figure 2E**). However, the percentage of PD-1^+^ EVs was uniformly low across all groups, including HDs (1.44 ± 1.63%), MM patients (3.28 ± 0.69%), NDMM (3.41 ± 0.83%), and RRMM (2.74 ± 1.21%), with no significant differences (**Figure 2F**). Among the checkpoint molecules we assessed, PD-L1 showed the highest expression and the most striking differences. As shown in **Figure 2G**, PD-L1 MRFI was significantly higher in MM-derived EVs (52 ± 14.1) compared to healthy controls (9.2 ± 2.15, p < 0.0001) with a marked increase in RRMM (78.66 ± 31.47) compared to NDMM (43.49 ± 15.69, p = 0.04). The percentage of PD-L1^+^ EVs was also significantly increased in MM patients (89.63 ± 2.17%) versus HDs (75.68 ± 11.46%, p = 0.001), with comparably high levels in NDMM (94.73 ± 2.8%) and RRMM (95.7 ± 0.75%) (**Figure 2H**). A representative experiment and flow cytometry profiles from three HD and three MM patients are shown in **Figures 3A, B**, respectively. Next, we analyzed the correlation between the expression of CD138 and immune checkpoints on MM-derived EV. A significant correlation was found between CD138 and HLA-G expression (p < 0.0001, **Figure 4A**), suggesting malignant PCs as a source of HLA-G+ immunosuppressive EVs. Conversely, no correlation was observed between CD138 and PD-1/PD-L1 (**Supplementary Figure 1**). To validate these data, we assessed the co-expression of CD138 and HLA-G. As shown in **Figure 4B**, we identified 10.3 ± 2.47% CD138^+^ EVs, 16.2 ± 2.53% HLA-G^+^ EVs, and 5.83 ± 2.25% double-positive CD138^+^/HLA-G^+^ EVs in patients, confirming that a considerable fraction of PCs-derived EVs carries HLA-G. To further validate these observations at the transcriptomic level, we analyzed HLA-G mRNA expression in PCs from patients and donors using the CoMMpass database. As shown in **Figure 4C**, HLA-G transcript levels were significantly higher in malignant PCs compared to other BM cell populations (log2(TPM+1), p = 0.0416), supporting the protein-level data and reinforcing the immunosuppressive phenotype of tumor cells. Overall, MM-derived EVs exhibit a distinct immune checkpoint profile, with markedly elevated levels of HLA-G and PD-L1. The correlation between CD138 and HLA-G suggests that malignant PCs-derived EVs actively contribute to immune evasion within the BM microenvironment. These findings highlight checkpoint-expressing EVs as potential biomarkers and therapeutic targets to restore anti-tumor immunity and overcome resistance in MM.

### 3.4 EVs from MM patients modulate immune responses

**Figure 5:**
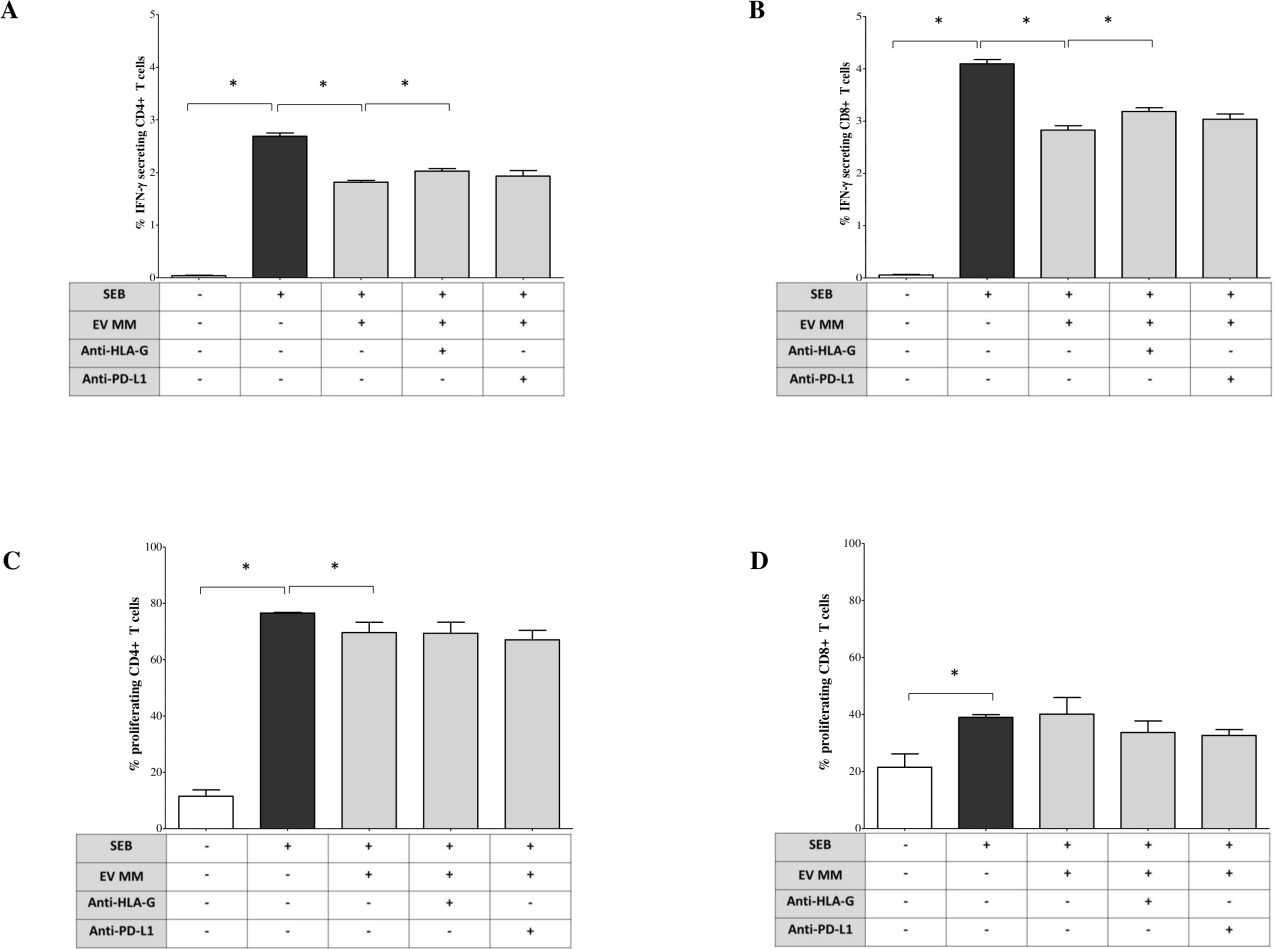
Immunoregulatory functions of BM-derived EV from MM patients. Peripheral blood mononuclear cells (PBMCs) from HDs were stimulated with Staphylococcal enterotoxin B (SEB, 1 μg/mL) in presence or absence of BM-derived EVs isolated from NDMM patients. A total of 1 × 10^9^ EVs per condition were added to each well. In selected conditions, blocking antibodies were included to assess the contribution of immune checkpoint molecules: anti–HLA-G (clone 87G, BioLegend) and anti–PD-L1 (clone 29E.2A3, BioLegend), each at a final concentration of 10 μg/mL, added at the time of stimulation. **(A, B)** Intracellular IFN-γ production by CD4^+^ and CD8^+^ T cells was evaluated after 18 hours of culture in the presence of brefeldin A, for the final 6 hours. Cells were then fixed, permeabilized, and stained for intracellular IFN-γ. Data are expressed as the median ± standard error (SE) of the percentage of IFN-γ^+^ cells among gated CD4^+^ **(A)** and CD8^+^ **(B)** T cells. **(C, D)** T cell proliferation: PBMCs labeled with CFSE before SEB stimulation and cultured for 6 days in the presence or absence of MM-derived EVs and/or blocking antibodies, FACS quantification for proliferating CD4^+^ **(C)** and CD8^+^ **(D)** T cells. Results are expressed as the median ± SE of the percentage of proliferating cells. Each experiment was performed using PBMCs from at least three independent healthy donors, and EVs from pooled MM BM samples were used to ensure consistency and reduce inter-patient variability. Statistical significance was assessed using paired or non-parametric tests as appropriate and is indicated in the figure (*p ≤ 0.05; **p ≤ 0.01; ***p ≤ 0.001; ns, not significant).

We next investigated whether EVs isolated from the BM plasma of MM patients modulate immune responses to the bacterial superantigen staphylococcal enterotoxin B (SEB). To assess early immune activation, we measured intracellular IFN-γ production in T lymphocytes from HDs post SEB stimulation of total MNCs, with or without the addition of MM-derived EVs. As shown in **Figure 5A**, SEB stimulation markedly increased the percentage of IFN-γ^+^ CD4^+^ T lymphocytes (median ± SE: unstimulated, 0.035 ± 0.01%; SEB-stimulated, 2.7 ± 0.06%). Co-incubation with MM-derived EVs significantly attenuated this response (1.83 ± 0.03%, p = 0.02). Importantly, the addition of an anti-HLA-G blocking antibody partially restored IFN-γ production (2.03 ± 0.04%, p = 0.05), suggesting a key role for HLA-G in mediating EV-induced immunosuppression. In contrast, the blockade of PD-L1 did not significantly rescue IFN-γ expression (1.91 ± 0.1%). Similar results were observed in CD8^+^ T lymphocytes (**Figure 5B**), where SEB stimulation increased the percentage of IFN-γ^+^ cells from a basal level of 0.06 ± 0.01% (unstimulated) to 4.11 ± 0.08%. This activation was significantly reduced by MM-derived EVs (2.88 ± 0.08%, p = 0.02) and partially restored by anti-HLA-G blockade (3.14 ± 0.07%, p = 0.05). Again, PD-L1 blockade had no significant effect (2.93 ± 0.09%). To assess the impact of EVs on later stages of the immune response, we evaluated T cell proliferation six days post-SEB stimulation. As shown in **Figure 5C**, SEB significantly enhanced CD4^+^ T cell proliferation (unstimulated, 10.3 ± 2.2%; SEB-stimulated, 76.5 ± 0.21%). The addition of MM-derived EVs led to a modest but significant reduction in cell proliferation (71.9 ± 3.6%, p = 0.02). In contrast, EVs had no suppressive effect on CD8^+^ T cell proliferation (**Figure 5D**) (unstimulated, 18.4 ± 4.6%; SEB, 39.4 ± 0.96%; SEB + EVs, 45.4 ± 5.8%). Notably, blocking HLA-G or PD-L1 did not restore CD4^+^ or CD8^+^ T cell proliferation in this late-phase setting. Overall, these results suggest that MM-derived EVs suppress early T cell activation, particularly IFN-γ production, and that HLA-G expressed on the surface of these EVs is partially involved in this immunosuppressive effect. However, EV effect on T cell proliferation at later stages appears limited. These findings support a model in which tumor-derived EVs contribute to early immune evasion, and suggest that targeting EV-associated HLA-G may represent a promising strategy to restore anti-tumor immunity in MM.

### 3.5 EVs from MM patients’ BM modulate cytokine secretion in SEB-stimulated MNCs

**Figure 6:**
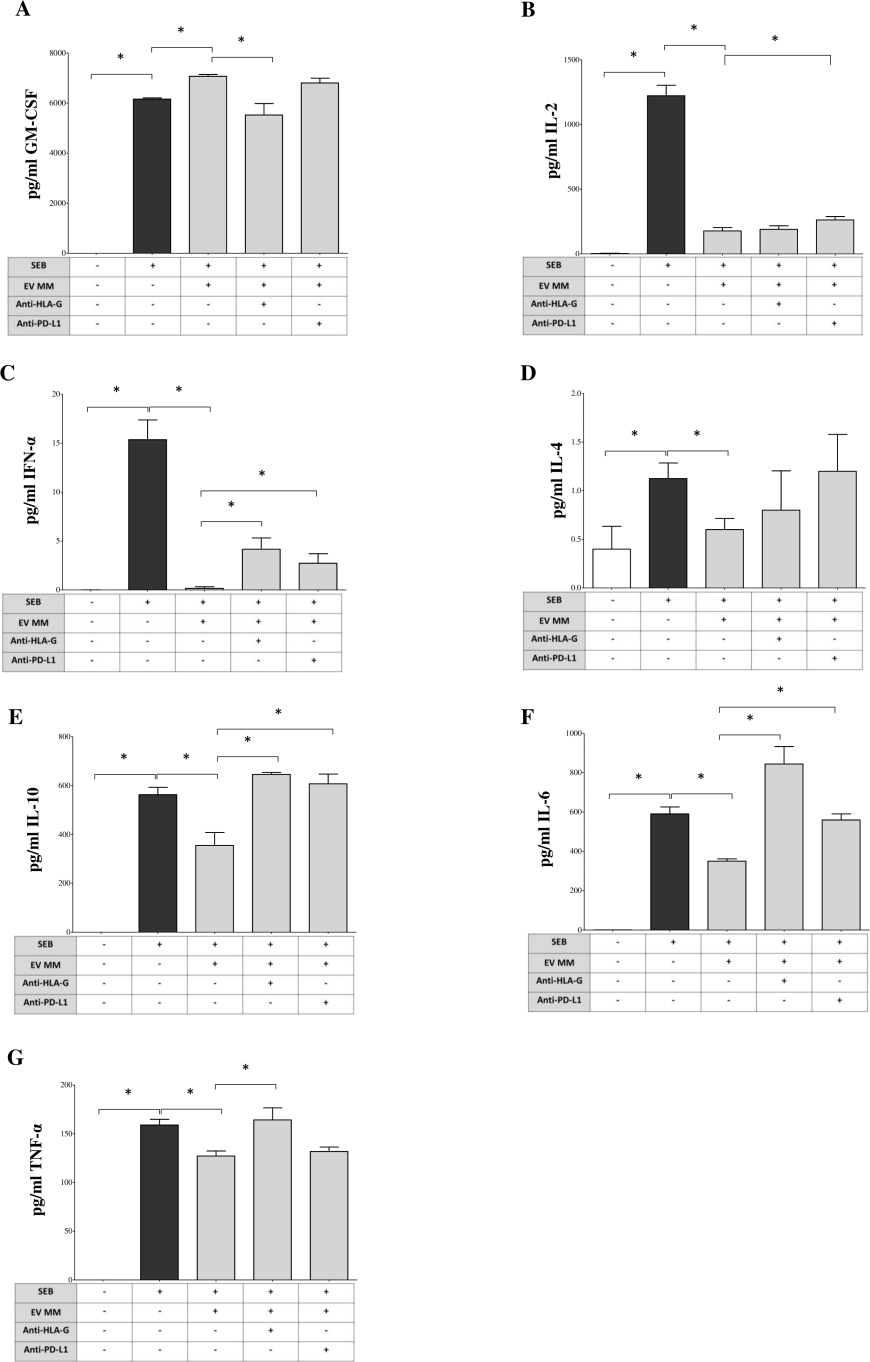
Modulation of cytokine production by BM-derived EVs from MM patients. PBMCs from HDs stimulated with SEB (1 μg/mL) and cultured for 6 days in the presence or absence of MM-derived BM-EVs and/or blocking antibodies against HLA-G (10 μg/mL, clone 87G) or PD-L1 (10 μg/mL, clone 29E.2A3). A total of 1 × 10^9^ EVs were added per condition. Supernatants were collected at day 6 and analyzed using a bead-based multiplex flow cytometry assay (Miltenyi Biotec) to quantify cytokine concentrations. The production of the following cytokines was assessed: GM-CSF **(A)**, IL-2 **(B)**, IFN-α **(C)**, IL-4 **(D)**, IL-10 **(E)**, IL-6 **(F)**, and TNF-α **(G)**. Results are expressed as pg/mL (median ± SE) from three independent experiments, each performed using PBMCs from different HDs and pooled EVs from MM patients. Statistical significance is indicated as follows: ns (not significant), *p ≤ 0.05, **p ≤ 0.01, ***p ≤ 0.001.

Next, we evaluated whether EVs derived from the BM plasma of MM patients modulate pro-inflammatory cytokine production by total MNCs. Again, we use SEB stimulation to induce cytokine release by MNCs. Cytokine concentrations in culture supernatants were measured under various experimental conditions, as previously described. As shown in **Figure 6A**, SEB induced a robust release of GM-CSF (pg/mL, median ± SE: 6154 ± 49.2 *vs*. 0 ± 0.2 unstimulated), which was further increased in the presence of MM-derived EVs (7119 ± 77.72, p = 0.02). This effect was significantly reduced by anti-HLA-G blockade (5660 ± 460.2, p = 0.05), while anti-PD-L1 had no impact (6888 ± 190.5). IL-2 secretion was also markedly induced by SEB (1297 ± 80.5 *vs*. 1.15 ± 2.1 unstimulated) and strongly suppressed by MM-derived EVs (184.5 ± 25.73, p = 0.05; **Figure 6B**). This inhibition was partially reversed by PD-L1 blockade (243.1 ± 25.93, p = 0.05), while anti-HLA-G had no significant effect (172.3 ± 27.53). IFN-α production was also significantly inhibited in EV-treated cultures compared to SEB alone (0 ± 0.16 *vs*. 15.35 ± 2.02, p = 0.02) (**Figure 6C**). Both anti-HLA-G (3.5 ± 1.1, p = 0.03) and anti-PD-L1 (3.1 ± 0.97, p = 0.03) partially restored IFN-α levels. For IL-4, SEB modestly increased secretion (1 ± 0.16 *vs*. 0.35 ± 0.23 unstimulated), reduced by exposure to MM-EVs (0.6 ± 0.11, p = 0.02; **Figure 6D**). However, neither checkpoint blockade significantly reversed this effect. Analysis of inflammatory and regulatory cytokines showed that SEB stimulation induced IL-10 (583 ± 30.29), IL-6 (617.2 ± 35.16), and TNF-α (153.7 ± 5.84) (**Figures 6E–G**, respectively). MM-derived EVs significantly suppressed all three cytokines: IL-10 (348.8 ± 52.76, p=0.02), IL-6 (360.7 ± 11.58, p=0.02), and TNF-α (132.2 ± 5.18, p = 0.02). These effects were reversed by anti-HLA-G, which restored IL-10 (646.5 ± 8, p = 0.05), IL-6 (839.6 ± 88.7, p = 0.03), and TNF-α (158 ± 12.41, p = 0.05). Anti-PD-L1 partially restored IL-10 (610.8 ± 39.87, p = 0.05) and IL-6 (565.9 ± 31, p = 0.03), but not TNF-α. No significant modulation was observed for IL-5, IL-9, IL-12p70, or IL-17A under any condition (data not shown). IFN-γ levels exceeded the upper detection limit of the assay across all conditions and are therefore not reported. Collectively, these results demonstrate that MM-derived EVs can profoundly alter cytokine responses in SEB-stimulated MNCs, with HLA-G playing a central role in suppressing key pro-inflammatory and immunoregulatory cytokines, and PD-L1 contributing more selectively. These findings reinforce the immunosuppressive role of EVs in the MM BM microenvironment and provide additional rationale for targeting EV-associated immune checkpoint molecules to restore effective anti-tumor immunity.

### 3.6 Clinical correlations

**Figure 7:**
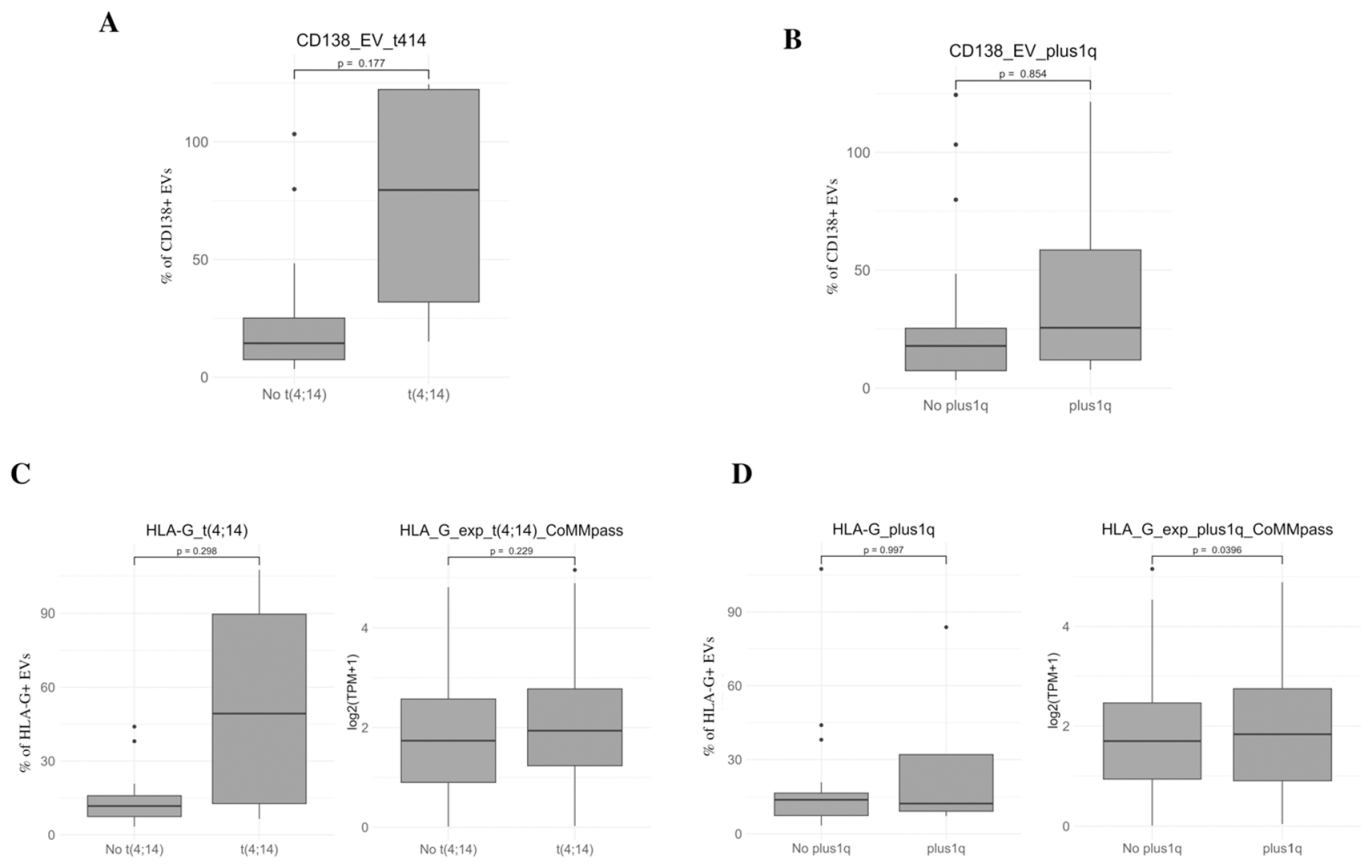
Clinical correlations of EV markers with cytogenetic abnormalities. **(A, B)** Percentage of CD138^+^ EVs in MM patients stratified by the presence or absence of high-risk cytogenetic alterations, including t(4;14) (left) and gain of 1q (1q^+^) (right) **(C, D)**. Expression of HLA-G on EVs (left panels) and HLA-G mRNA levels in CD138^+^ plasma cells from the CoMMpass dataset (right panels) in patients stratified by t(4;14) **(C)** and 1q gain **(D)**. Protein-level results are expressed as percentage of HLA-G^+^ EVs, while transcript levels are shown as log_2_ (TPM + 1). Box plots display the median, interquartile range (IQR), and minimum/maximum values. Statistical significance was assessed using appropriate non-parametric tests and the corresponding p value is indicated.

As no significant associations were observed between EV parameters and clinical outcomes (data not shown), likely due to the limited sample size, we redirected our focus toward biological features, specifically examining potential correlations with key cytogenetic abnormalities. We evaluated the expression of CD138 and HLA-G on EVs, as well as HLA-G transcript levels, in relation to high-risk alterations such as t(4;14) and gain of 1q (plus1q). As shown in **Figure 7A**, patients harboring the t(4;14) translocation exhibited a trend toward higher percentages of CD138^+^ EVs, compared to those without the abnormality, although this difference did not reach statistical significance (p = 0.177). Similarly, CD138^+^ EV levels were comparable between patients with or without gain of 1q (p = 0.854). We next analyzed HLA-G protein expression on EVs and HLA-G mRNA levels in malignant plasma cells using the CoMMpass dataset, stratified by cytogenetic risk groups. As shown in **Figure 7B**, patients with t(4;14) also showed a non-significant trend toward increased HLA-G expression at both the protein (EV) and transcriptomic levels (p = 0.298 and p = 0.229, respectively). Interestingly, while HLA-G expression on EVs did not differ between patients with or without 1q gain (p = 0.997), HLA-G transcript levels were significantly higher in patients with 1q+ (p = 0.0396). Taken together, these findings suggest that high-risk cytogenetic features, particularly gain of 1q, may be associated with increased expression of immunosuppressive molecules such as HLA-G at the mRNA level, potentially contributing to an immune-evasive phenotype in MM. Although most associations did not reach statistical significance, the consistent trends observed, especially in the context of known high-risk genomic alterations, underscore the need for larger, well-powered studies to validate these preliminary observations and to further elucidate the role of EVs in MM immune regulation.

## 4 Discussion

Although initially considered marginal players in cancer biology, EVs are now recognized as important contributors to tumor progression. Over the past decade, mounting evidence has firmly established EV involvement in key oncogenic processes across both solid and hematologic malignancies (25, 26). As a result, EVs have emerged as valuable biomarkers for diagnosis and disease monitoring, and increasingly as potential therapeutic targets (27, 28). By mediating intercellular communication, tumor-derived EVs actively shape the tumor microenvironment, facilitating immune escape, supporting metastasis, and promoting drug resistance (19, 26). We previously reported the immunoregulatory and anti-inflammatory functions of BM-derived EVs in neuroblastoma, mediated by expression of HLA-G and PD-L1 (29). Such results advocated for plausible similar mechanisms in myeloma, where EVs have already been implicated in key pathological processes such as modulation of the BM microenvironment (30), leading to metastatic (31) and pathological processes such as angiogenesis and osteolysis (32–35). In addition, the concentration of circulating exosomes is significantly increased in MM patients compared to individuals with MGUS and healthy controls, as previously reported using plasmonic biosensing technologies (36). Furthermore, MM-derived EVs are known to carry surface ligands for inhibitory immune receptors, suggesting a broader role in immune modulation (37, 38). However, despite these insights, a systematic evaluation of their immunosuppressive functions in MM has remained limited.

Mechanistically, EVs retain membrane components from cells of origin, enabling them to interact with immune cells and suppress effector functions, most notably by inhibiting T cell and NK cell activity. This concept is supported by the identification of HLA-G^+^ EVs in various tumor types, where their presence correlates with disease progression (39–44). Similarly, PD-L1^+^ EVs have been detected across multiple cancers (45–58), with their abundance associated with clinical outcomes and responses to CAR-based therapies and immune checkpoint blockade (50–52, 57, 59–64). Building on this evidence, we measured BM-derived EVs from MM patients expressing significantly higher levels of HLA-G, PD-L1, and PD-1, compared to healthy subjects. To our knowledge, this is the first report of HLA-G^+^ EVs in the BM of MM patients. Such findings broaden mechanistic knowledge on EV-mediated immune checkpoint regulation in MM, and the results here described complement previous studies where the role of soluble HLA-G in tumor burden and disease progression was suggested as a potential biomarker for disease monitoring and risk stratification (7, 65). While PD-L1^+^ EVs have previously been described, particularly in patients receiving anti-CD38 monoclonal antibody therapy such as daratumumab (66, 67), our findings extend this observation to treatment-naïve patients, highlighting the relevance of EV-mediated immune suppression across disease stages. To gain insight into the cellular origin of these checkpoint-expressing EVs, we assessed tumor-associated antigens, with the detection of CD138 supporting a malignant plasma cell origin for a subset of EVs. However, our immunophenotypic analysis revealed that monocytes, NK cells, and potentially MSC also contribute to the EV pool. Notably, PD-1^+^ EVs may be primarily derived from monocytes, whose contribution to EV release was demonstrated by a high percentage of CD14+ EVs. NK cells, despite their cytotoxic potential, may instead play a regulatory role in limiting EV release, consistent with their established anti-tumor functions. Interestingly, we observed no significant differences in the expression of immune checkpoint molecules between EVs from newly diagnosed and relapsed/refractory MM patients, suggesting that these immunosuppressive features are established early in disease progression. However, the limited follow-up duration in our cohort precluded a definitive assessment of their prognostic significance. In support of a potential link between EV-mediated immune suppression and MM pathobiology, we observed that patients with high-risk cytogenetic alterations, particularly gain of chromosome 1q, exhibited significantly higher HLA-G expression at the mRNA level in malignant plasma cells. This association suggests that immune-evasive mechanisms involving HLA-G may be more prominent in genomically aggressive disease, and that EV-associated checkpoints could serve as both functional and molecular markers of high-risk MM. Functionally, our data demonstrate that MM-derived EVs impair key aspects of T cell immunity, reinforcing their role as active participants in immune evasion. EVs significantly reduced IFN-γ production by both CD4^+^ and CD8^+^ T cells, an effect primarily mediated by HLA-G, as PD-L1 blockade had little impact. Given the central role of IFN-γ in orchestrating anti-tumor responses (68), this finding underscores the immunosuppressive capacity of HLA-G–expressing EVs. While we initially attempted to quantify secreted IFN-γ using a multiplex assay, technical limitations, such as signal saturation, and the inability to dilute samples without compromising detection of other cytokines, prevented reliable measurement. To overcome such limitations, we plan to implement omic-based experiments in future analyses to implement current results with complementary insights. In contrast, T cell proliferation was only modestly affected, with slight reductions in CD4^+^ cells. Notably, blocking HLA-G or PD-L1 did not restore proliferative capacity, implicating additional immunoregulatory pathways, such as adenosinergic signaling, in EV-mediated suppression, as previously reported (22). Beyond direct effects on T cells, MM-derived EVs altered cytokine secretion profiles in SEB-stimulated MNCs. They enhanced GM-CSF production, partially via HLA-G, which may promote the recruitment of tumor-associated neutrophils and macrophages and induce PD-L1 expression on these cells (69, 70). Conversely, IFN-α and IL-6 secretions were suppressed. While IFN-α has known anti-tumor effects and is used therapeutically in MM (71), IL-6 plays a dual role, both supporting tumor growth and stimulating T cell activity (72). Partial restoration of these cytokines upon checkpoint blockade supports a direct role for HLA-G and PD-L1 in modulating cytokine responses. Additional cytokines, including IL-2, IL-4, IL-10, and TNF-α, were also influenced. Among these, IL-2 and TNF-α are especially relevant for T cell expansion and anti-tumor immunity (73, 74), while IL-4 and IL-10, linked to Th2 responses, may play a lesser role in MM immune surveillance. The incomplete reversal of cytokine suppression by checkpoint inhibition suggests involvement of other immunosuppressive mediators, such as adenosine and additional inhibitory ligands (22, 37).

In conclusion, this study provides a comprehensive characterization of BM-derived EVs in MM, highlighting their expression of key immune checkpoint molecules and their ability to suppress T cell function and modulate cytokine networks. We demonstrated that HLA-G and PD-L1 are partially involved in EV-mediated immunosuppression, qualifying them as potential therapeutic targets. Moreover, the observed association between HLA-G expression and high-risk cytogenetic features, particularly 1q gain, suggests that EV-driven immune regulation may be linked to more aggressive disease biology. Future studies are warranted to explore the prognostic relevance of checkpoint-expressing EVs and to evaluate whether targeting EV-associated immune checkpoints can restore anti-tumor immunity and improve clinical outcomes in MM.

## Data Availability

The raw data supporting the conclusions of this article will be made available by the authors, without undue reservation.
