# Supplementary figures and images for: Bone marrow-derived extracellular vesicles from multiple myeloma patients promote adaptive immune dysfunction via HLA-G, PD-1, and PD-L1
Source: Front Immunol. 2025 Aug 28;16:1640168. doi: 10.3389/fimmu.2025.1640168 (PMC12422937; doi:10.3389/fimmu.2025.1640168)

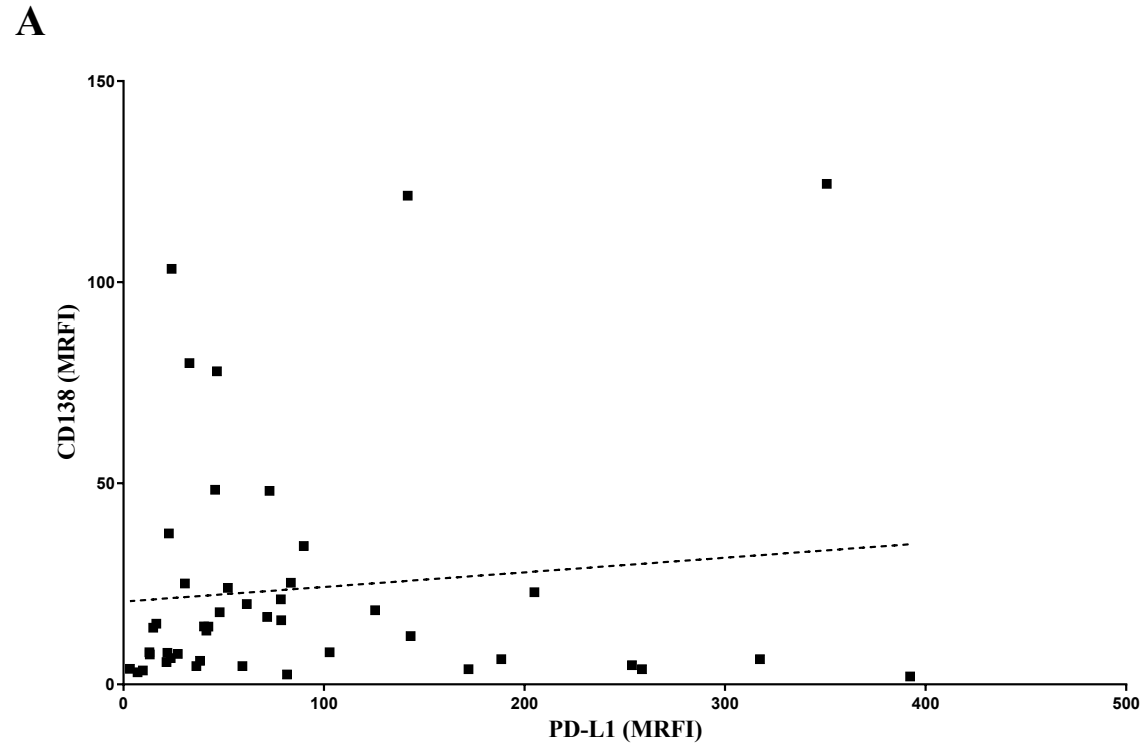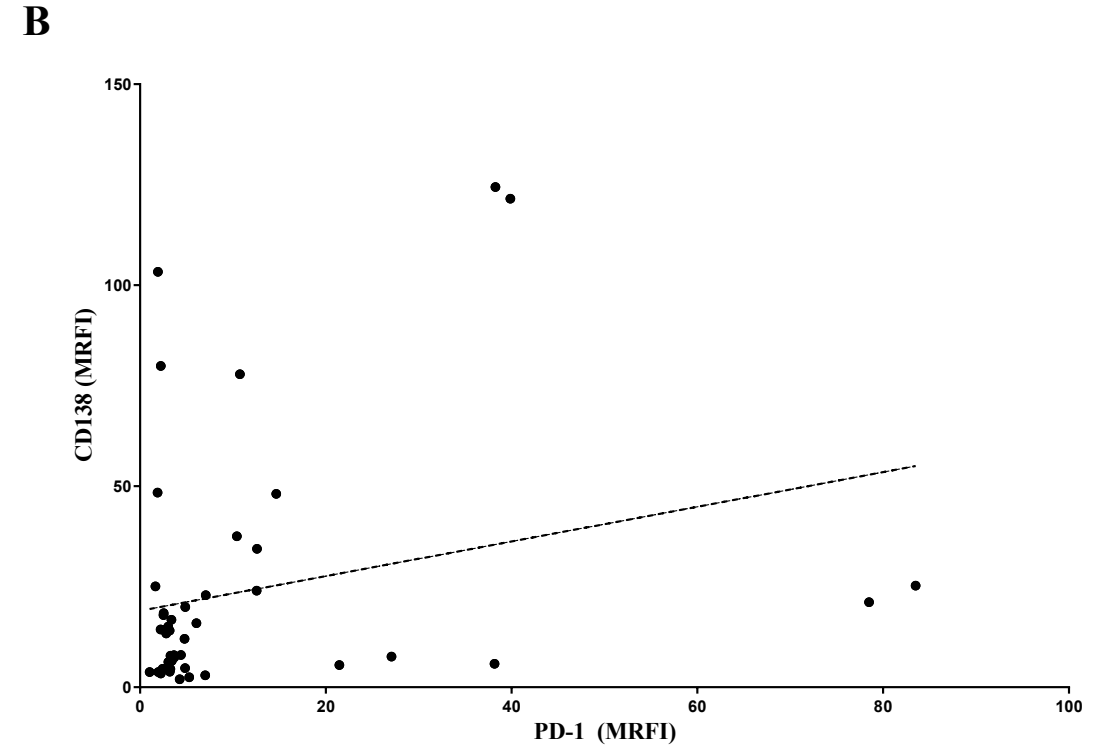

Correlations and linear regressions between CD138 and PD-L1 (panel A) or PD-1 (panel B).

Supplement: Supplementary file 1 [file DataSheet1.pdf]
